# KSHV gB associated RGD interactions promote attachment of cells by inhibiting the potential migratory signals induced by the disintegrin-like domain

**DOI:** 10.1186/s12885-016-2173-9

**Published:** 2016-02-24

**Authors:** Hosni A. M. Hussein, Lia R. Walker, Shaw M. Akula

**Affiliations:** Department of Microbiology & Immunology, Brody School of Medicine at East Carolina University, Greenville, NC 27834 USA

**Keywords:** Integrins, Disintegrins, RGD, DLD, KSHV gB, Migration

## Abstract

**Background:**

Kaposi’s sarcoma-associated herpesvirus (KSHV) glycoprotein B (gB) is not only expressed on the envelope of mature virions but also on the surfaces of cells undergoing lytic replication. Among herpesviruses, KSHV gB is the only glycoprotein known to possess the RGD (Arg-Gly-Asp) binding integrin domain critical to mediating cell attachment. Recent studies described gB to also possess a disintegrin-like domain (DLD) said to interact with non-RGD binding integrins. We wanted to decipher the roles of two individually distinct integrin binding domains (RGD versus DLD) within KSHV gB in regulating attachment of cells over cell migration.

**Methods:**

We established HeLa cells expressing recombinant full length gB, gB lacking a functional RGD (gBΔR), and gB lacking a functionally intact DLD (gBΔD) on their cell surfaces. These cells were tested in wound healing assay, Transwell migration assay, and adhesion assay to monitor the ability of the RGD and DLD integrin recognition motifs in gB to mediate migration and attachment of cells. We also used soluble forms of the respective gB recombinant proteins to analyze and confirm their effect on migration and attachment of cells. The results from the above studies were authenticated by the use of imaging, and standard biochemical approaches as Western blotting and RNA silencing using small interfering RNA.

**Results:**

The present report provides the following novel findings: (i) gB does not induce cell migration; (ii) RGD domain in KSHV gB is the switch that inhibits the ability of DLD to induce cellular migration thus promoting attachment of cells.

**Conclusions:**

Independently, RGD interactions mediate attachment of cells while DLD interactions regulate migration of cells. However, when both RGD and DLD are functionally present in the same protein, gB, the RGD interaction-induced attachment of cells overshadows the ability of DLD mediated signaling to induce migration of cells. Furthering our understanding of the molecular mechanism of integrin engagement with RGD and DLD motifs within gB could identify promising new therapeutic avenues and research areas to explore.

**Electronic supplementary material:**

The online version of this article (doi:10.1186/s12885-016-2173-9) contains supplementary material, which is available to authorized users.

## Background

One of the hallmarks of cancer progression is cell attachment, migration, and invasion [1–3]. All of these key features are mediated by interactions with integrin-associated signaling [4]. The most commonly described integrins are those that recognize a small tripeptide amino acid sequence, RGD (Arg-Gly-Asp), and effectively mediate cell attachment, migration, and invasion [5, 6]. Of late, a small group of proteins referred to as disintegrins has also been described to bind integrins [7]. Disintegrins are non-enzymatic polypeptides broadly distributed in the venoms of viperid snakes, which antagonize the functions of several types of integrin receptors [8]. The minimum component of the disintegrin module required for integrin engagement is the 12- to 13-amino-acid disintegrin loop, for which a consensus sequence has been described: RX_6_DLXXF [9]. We hypothesized that RGD and disintegrin-like domain (DLD) had distinct roles to play in regulating attachment and migration of cells.

We employed Kaposi’s sarcoma-associated herpesvirus (KSHV) encoded glycoprotein B (gB) in functional assays to test our hypothesis. KSHV gB possess both of the aforementioned integrin binding domains. Among the α, β, and γ herpesvirus gB sequenced to date, KSHV gB possesses not only the RGD motif but also a functional disintegrin-like domain (DLD) [10]. Both RGD (27-29*aa*) and DLD (66-85*aa*) are placed juxtaposed in the *N-terminus* ectodomain region of the gB. In the case of KSHV gB, the DLD sequence is RX_5-7_D/**E**LXXF/LX_5_C (66-85aa; with a conservative D to **E** substitution). KSHV gB is not only expressed on the viral envelope but also on the infected cell membranes [11]. Earlier studies established the fact that soluble form and membrane associated full length gB could mediate cell attachment to extracellular matrix protein (ECM)-coated wells or a matrigel via binding to RGD-binding integrins [12]. In the present study, we have attempted to answer the following questions: (i) Does gB, a protein that possess both RGD and DLD mediate migration of cells? (ii) What are the distinct roles of RGD and DLD in promoting attachment and migration of cells? We concluded that the RGD and DLD interactions with integrins have distinct roles in affecting the function of a protein. Our study, for the first time describes RGD domain as a switch that regulates function of DLD contained within the same protein (gB) to effectively assist attachment of cells versus migration. A short discussion on how these divergent integrin-based interactions will alter KSHV pathogenesis is also provided.

## Methods

### Cells

A human cervical cancer HeLa cell line, human umbilical vein endothelial cells (HUVEC; Invitrogen, Carlsbad, CA), and *Spodoptera frugiperda* ovarian cells (Sf9) were propagated as per standard laboratory procedures [10, 13, 14].

### Transfection of cells and silencing PIKfyve RNA (SiRNA)

To establish stably transfected HeLa cells expressing different recombinant gB and gH proteins, cells (5x10^5^ cells) were seeded onto 24 well plates. Post 24 h of seeding, the cells were transfected with the respective plasmid DNA using FuGENE HD transfection reagent (Promega, Madison, WI). These cells were cultured in selection medium containing 500 μg/ml of G418 from the second day of transfection for a duration of 8 weeks after which the expression of genes encoding different gB proteins were confirmed by flow cytometry and RT-PCR. At least 2 pools of cells/each plasmid that were under the selection for about 8 weeks were tested in our experiments. Expression of PIKfyve was inhibited by the transfection of HeLa cells which were stably transfected to express gBΔR with double-stranded RNA oligonucleotides as described previously [15, 16]. The PIKfyve siRNAs used in this experiment were obtained from GE Healthcare, Dharmacon RNAi & Gene expression (Lafayette, CO) as the ON-TARGET plus Smart pool [17]. The nonspecific (NS) siRNAs used were those described previously [18]. Efficiency of silencing the gene was confirmed by performing Western blotting at 48 h post transfection using specific antibodies.

### Antibodies, inhibitors, and soluble proteins

An antibody to DLD peptide sequence of gB (anti-DLD) [10], rabbit antibodies to the RGD-containing sequence of gB (anti-RGD) [19], rabbit antibodies to the C-terminal domain in gB (anti-gB-C) [19], rabbit anti-gB antibodies [11], and rabbit anti-gH antibodies [20] were used in this study. Polyclonal sheep antibodies to PIKfyve (R&D systems, Minneapolis, MN) and polyclonal rabbit antibodies to β-actin (Cell Signaling, Beverly, MA) were used in the Western blotting experiments. Cytochalasin D (Cyto-D) and Rac-1 inhibitor, NSC23766, purchased from Sigma-Aldrich, St. Louis, MO were used in this study. His-tagged, recombinant and soluble KSHV gBΔTM [21], gBΔTM lacking the RGD (gBΔTM-RGA; referred to as gBΔTMΔR) [21], and gBΔTM lacking the DLD (gBΔTMΔD) [10] were expressed and purified from Sf9 cells as per earlier studies [10]. Vascular endothelial growth factor (VEGF) purchased from R&D Systems [18] was used as a positive control in MTT assay.

### Cloning of full length forms of recombinant gB

Clones gB/pCDNA3.1(+), gBΔR/pCDNA3.1(+), and gBΔD/pCDNA3.1(+) encoding the full length gB (2535 bp), gB lacking a functional RGD, and a functional DLD, respectively, were used in this study. Clone gBΔR mutant was generated by mutating one of the existing RGD amino acids (GAAHS**RGD**TFQTS; RGD sequence is in bold) of KSHV gB to alanine (GCG) (GAAHSRG**A**TFQTS; alanine point mutation is bolded). The point mutations within the RGD sequence of KSHV gB were achieved by using the gB/pCDNA3.1(+) plasmid as the dsDNA template and appropriate primers: RGA.F forward (5’-GCGGCGCACTCGAGGGGT**GCC**ACCTTTCAGACGTCCAGTT-3′; bolded region depicts the alanine point mutations to RGD, coding strand) and RGA.R reverse (5′-AACTGGACGTCTGAAAGGT**GGC**ACCCCTCGAGTGCGCCGC-3′; bolded region depicts the alanine point mutations to RGD, non-coding strand); along with the QuikChange XL site-directed mutagenesis kit (Stratagene, La Jolla, CA) to yield the gBΔR/pCDNA3.1(+). Clone gBΔD/pCDNA3.1(+) was generated as described above and using primers described in our earlier study [10].

### Flow cytometry

Flow cytometry was used to monitor expression of gB and gH on the cells. The trypsinized transfected cells were washed, incubated in growth medium at 37 °C for 30 min, centrifuged and resuspended in cold PBS. The entire procedure was performed at +4 °C. Cells were incubated with different antibodies at 4 °C for 30 min, washed, incubated with FITC conjugated appropriate secondary IgG at 4 °C for 30 min, washed and analysed in a FACScan flow cytometer (Becton Dickinson) with appropriate gating parameters.

### In vitro transcription and translation

The IVT of different plasmids was performed using the [^35^S]methionine and TNT-coupled rabbit reticulocyte lysate system (Promega, Madison, WI) with canine pancreatic microsomal membranes according to the manufacturer's recommendations [11].

### Wound healing migration assay

Untransfected, HeLa cells expressing different forms of gB or gH were grown to 80–90 % confluence in a 1 % collagen coated 24-well plates. The monolayers of cells were wounded by performing a scratch with a sterile 1000 μl micropipette tip as described earlier [22]. Cells were washed with DMEM, and further incubated at 37 °C in DMEM containing 2 % FBS. Wound closure was monitored at 16 h post scratch and imaged with a laser-scanning LSM 510 Carl Zeiss confocal microscope (Magnification, x 20 objective). The open area (scratch) was quantified with TScratch software [23].

### Transwell migration assay

A three-dimensional cell migration assay was performed using A Transwell 8-mm permeable membrane insert coated with a 1 % collagen solution (Millipore Corp., Billerica, MA). HeLa cells expressing various recombinant gB proteins were cultured for 24 h in serum-free medium. A 100 μl of 1x10^6^ cells/ml suspension was added to the upper chamber of 24-well Transwell plates and complete DMEM medium (containing10% FBS) was added to the bottom chamber and incubated at 37 °C. At the end of 16 h incubation, the non-migrated cells on the upper face of the membrane were removed by using a sterile cotton swab and migrated cells on the lower face were fixed in paraformaldehyde, permeabilized by methanol (100 %) for 10 min, stained with crystal violet for 30 min and quantitated by microscopy [24].

### Cell adhesion assay

Cell adhesion assays were performed as per earlier methods [21]. Maxisorp 96-well immune plates (Thermo Scientific, Waltham, MA) were coated with 100 μl of different concentrations of soluble proteins overnight at 4 °C in PBS. After three washes with sterile PBS, plates were blocked with 1 % BSA in PBS for 2 h at 4 °C and washed three times. HeLa cells were trypsinized and seeded into the protein-coated wells of the plate at a concentration of 2x10^4^ cells/well in a 100 μl volume. The plate contents were incubated at 37 °C in a 5 % CO2 atmosphere with 100 % humidity for 1 h and washed four times with serum-free DMEM, and the adherent cells were fixed with 4 % paraformaldehyde in PBS for 30 min at room temperature. Adherent cells were washed and stained with 0.5 % crystal violet in water with 20 % (vol/vol) methanol for 15 min at room temperature. After 15 min, the cells were extensively washed in PBS, dye was extracted with 0.1 M sodium citrate and quantified by the measurement of absorbance at 595 nm in an ELISA plate reader. In another set of experiments, we incubated protein-coated wells of the plate with respective antibodies to different domains of gB prior to seeding cells and monitoring the adhesion property as per above procedures.

We also performed the above described cell adhesion assays with HeLa cells expressing gB, gBΔR, and gBΔD on their cell membranes. The ability of membrane associated gB to support adhesion of cells was also confirmed by incubating these cells with specific antibodies to different integrin binding domains contained within gB for 1 h at +4 °C prior to conducting the adhesion assay using collagen coated wells.

### Monitoring dynamic events on the cell membranes

Target cells were washed in phosphate buffered saline (PBS) and fixed with 3.7 % formaldehyde in PBS for 10 min. The cells were permeabilized with 0.1 % Triton X-100 in PBS for 3 min, washed in PBS, and blocked in PBS containing 1 % bovine serum albumin (BSA). These cells were stained with rhodamine-labeled phalloidin (Biotium, Hayward, CA) diluted (as per the manufacturer’s recommendations) in PBS for 20 min at room temperature before being washed, mounted using SlowFade Antifade Reagent containing DAPI (Life Technologies, Grand Island, NY), and examined under Nikon fluorescent microscope using appropriate filters. The number of filopodial structures on the individual cells observed under confocal microscope was counted manually as described in earlier studies [25]. In another set of experiments, permeabilized cells were sequentially stained with mouse anti-Rac1 IgG2b monoclonal antibodies (Thermo Scientific) and FITC-labeled appropriate secondary antibodies as per our earlier studies [26]. Following this procedure the cells were further stained with rhodamine-labeled phalloidin as described above. This was conducted to appreciate the distribution of Rac1 in cells expressing different forms of gB in terms of filopodial extensions.

### Measuring active Rac1 by pull-down with GST-Pak1

Rac1/Cdc42 activation assay was done using the Active Rac1/Cdc42 Pull-Down and Detection kit (Thermo Scientific Pierce). The assay employs a GST-fusion protein containing the p21-binding domain of p21-activated protein kinase 1 (PAK1) to selectively bind active Rac1/Cdc42 in whole cell lysates. The active Rac1/Cdc42 from different cell lysates was determined as per the manufacturer’s recommendation. Briefly, the active Rac1/Cdc42 bound to the glutathione agarose beads was quantified by immunoblot analysis using anti-Rac1 and anti-Cdc42 monoclonal antibody (Thermo Scientific Pierce). The value for active Rac1/Cdc42 collected on the beads was normalized to the concentration of protein in the cell lysate. On a separate immunoblot, the total Rac1 and Cdc42 in a sample of the cell lysate was determined and normalized to the protein concentration. The normalized results were then expressed as the ratio of active Rac1/Cdc42 to total Rac1/Cdc42 in the sample.

### Cell proliferation assay

Cell proliferation was monitored using the 3-(4,5-dimethylthiazol-2-yl)-2,5-diphenyltetrazoliumbromide (MTT) assay that was performed according to standard procedures [27]. Each experiment was repeated three times. Cell proliferation was also conducted by the conventional cell counting technique as per standard protocols [28].

## Results

### Generating clones of *orf8* to express full length gB with mutations to DLD and RGD

**Fig. 1:**
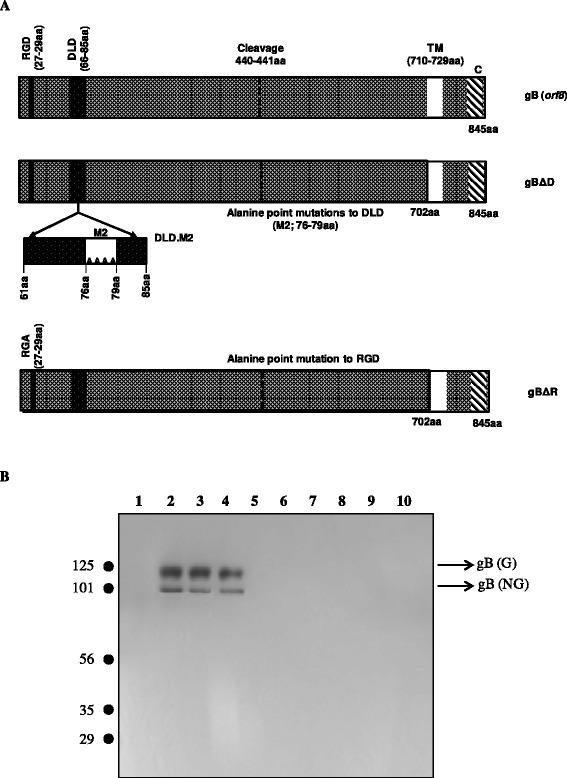
Generating KSHV gBΔD. **a** The diagram shows the schematic of *orf8* encoding the full length gB (gB/pCDNA3.1) compared to gB lacking a functional DLD (gBΔD/pCDNA3.1) and RGD (gBΔR/pCDNA3.1), respectively. **b** *In vitro* transcription and translation of KSHV *orf8*. IVT was performed using pCDNA3.1 (*lane 1, 6*), gB/pCDNA3.1 (*lane 2, 7*), gBΔD/pCDNA3.1 (*lane 3, 8*), gBΔR/pCDNA3.1 (*lane 4, 9*), and gH/pCDNA3.1 (*lane 5, 10*) using the microsomal membranes. The IVT products were immunoprecipitated using either rabbit anti-gB antibodies (*lanes 1 – 5*) or pre-immune IgGs (*lanes 6 – 10*). The immunoprecipitates were resolved on a 10 % SDS-PAGE gels and autoradiographed. The letters ‘G’ and ‘NG’ denote glycosylated and non-glysoylated forms of gB

Full length KSHV *orf8* is 2535 bp long that encodes a 845aa gB [29]. In earlier studies we had described the generation of the full length gB protein and its characterization [11]. In the present study, we used the gB/pCDNA3.1(+) clone [11] to generate full length gB lacking the RGD (gBΔR/pCDNA3.1) and DLD (gBΔD/pCDNA3.1) domains (Fig. 1a). The gBΔR possess a RGA instead of RGD motif while gBΔD possess a RVCSASITGEAAAANLEQTC instead of RVCSASITGELFRFNLEQTC motif. The authenticity of the generated clones was confirmed by sequencing. This was a crucial step to characterize a role for DLD and RGD of gB in mediating cell attachment and migration. The clones of gB/pCDNA3.1, gBΔR/pCDNA3.1, gBΔD/pCDNA3.1, and gH/pCDNA3.1 were *in vitro* transcribed and translated (IVT) in the presence of the canine microsomal membranes. Rabbit anti-gB antibodies were used to immunoprecipitate gB from the IVT products. Rabbit anti-gB antibodies specifically immunoprecipitated both the glycosylated (126 kDa) and the non-glycosylated (94 kDa) forms of the full length gB, gBΔR, and gBΔD (Fig. 1b; *lanes* 2-4). Rabbit anti-gB antibodies did not react significantly with IVT products generated using empty vector (Fig. 1b; *lane 1*) and gH/pCDNA3.1 (Fig. 1b; *lane 5*). Pre-immune IgGs failed to immune precipitate gB from any of the IVT products generated (Fig. 1b, *lanes* 6-10). These results demonstrated the authenticity of the gB clones generated as well as the specificity of the rabbit anti-gB antibodies to immunoprecipitate gB from the IVT products.

### Full length gB lacking a functional RGD domain promotes cell migration

Ideally, human microvascular endothelial cells-dermal (HMVEC-d) would have been used in this study. Unfortunately, we could not use them for the following three reasons: (i) in our hands, the transfection efficiency observed was poor [30]; (ii) these primary cells did not do well upon performing the scratch (wound) compounded by growing them in medium containing 2 % FBS; (iii) these cells do not support repeated passages under selection conditions [31]. In order to test the ability of gB to mediate cell migration, we utilized adherent HeLa cells [32] primarily because these cells have been extensively used to study cell migration compared to HMVEC-d, HFF and 293 cells [33, 34].

**Fig. 2:**
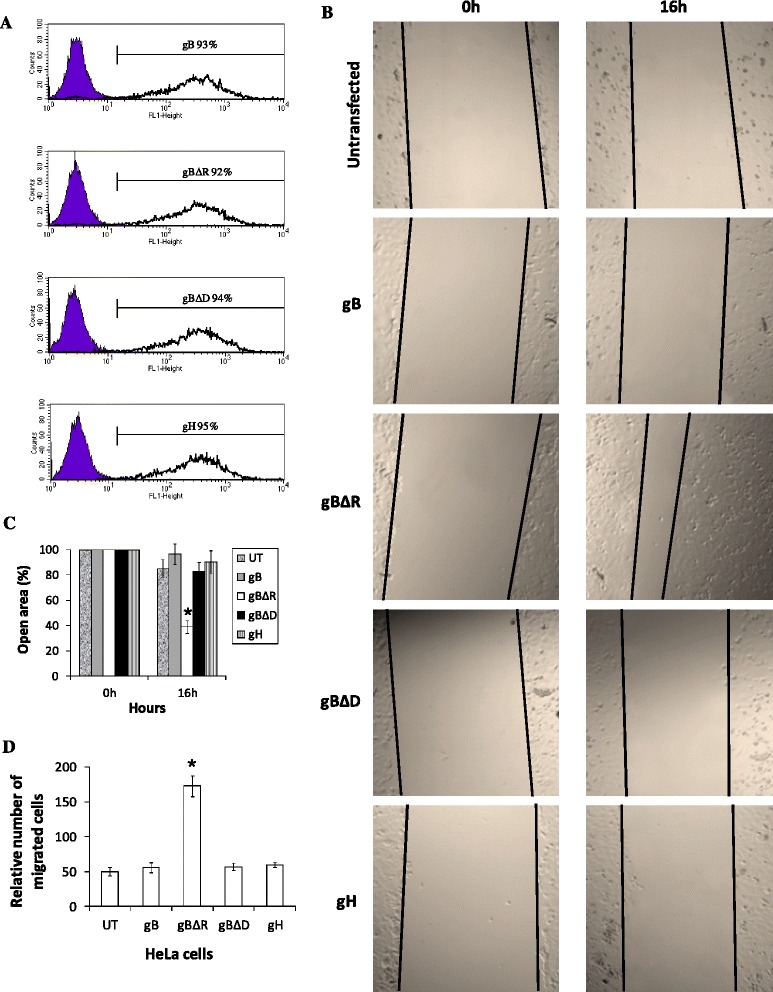
Expression of gBΔR accelerates cell migration in wound healing assays. **a** HeLa cells stably expressing gB, gBΔR, gBΔD, and gH were analyzed for the surface expression of gB and gH (respectively). This was performed by staining with pre-immune IgG (shaded purple), rabbit polyclonal anti-gB antibodies (black outline), or rabbit polyclonal anti-gH antibodies (black outline) followed by incubation with goat anti-rabbit FITC, before examining by FACS. The average percentage number of cells positive for the surface expression of gB and gH from three independent experiments is provided over the marker. A representative histogram plot for each cell type is depicted. **b** Untransfected HeLa cells or cells expressing gB, gBΔD, gBΔR, and gH in 24 well plates when 80-90 % confluent were scratched with a 1000 μl pipette tip. Wound closure was monitored at 16 h post scratch and imaged with a laser-scanning LSM 510 Carl Zeiss confocal microscope (Magnification, x 20 objective). A representative image of cell migration is provided. **c** All the wound healing assays performed in this study were independently repeated five times. The open area (scratch) was quantified with TSratch software and the data represented as a histogram. **d** Expression of gBΔR on the surface of cells induces directional migration. Data from Transwell migration assays showing alterations on the migration of HeLa cells through a permeable membrane in response to expression of various recombinant gB is depicted. Columns with asterisk mark (panels **c** and **d**) denotes the value to be statistically significant (*p* < 0.05) by least significant difference (LSD)

HeLa cells were transfected with plasmids encoding full length gB, gB lacking a functional RGD (gBΔR), gB lacking a functional DLD (gBΔD), and gH. We used gH as a control in this study; it is another glycoprotein like gB encoded by KSHV, lacking a functional RGD motif. These transfected cells were cultured with selection medium for 2 months to establish HeLa cells stably expressing different forms of recombinant proteins. The recombinant proteins were expressed on 90–95 % of cells (on the surfaces) as monitored by flow cytometry. Representative plots are provided showing the expression of virus encoded different forms of gB and gH on the surface of cells as determined by flow cytometry (Fig. 2a). These cells were tested in wound healing assays performed using collagen coated wells. The cells were induced to migrate into a wound created by scratching confluent cultures with a pipette tip to analyze the effect of RGD and DLD domains contained within the gB in promoting migration of cells. The open area was rapidly covered in cells expressing gBΔR compared to those untransfected (UT) or cells expressing gB, gBΔD, or gH (Fig. 2b). Cells expressing empty vector did not significantly promote cell migration (data not shown). Quantification of the wound closure is represented by the bar diagram in Fig. 2c. These results were based on monitoring cell migration at 16 h post scratch. The above results were confirmed on separate dates using an alternate pool of cells expressing different recombinant proteins (data not shown). Also, identical results were observed in transiently transfected HUVEC cells (Additional file 1: Figure S1b). We also monitored cell migration at 8–24 h post scratch. The difference in cell migration between different treatments was apparent by 12 h post scratch and was optimal by 16 h post scratch. By 20-24 h post scratch, the wound in cells expressing gBΔR was completely closed compared to cells expressing other constructs (Additional file 1: Figure S1a). The results from wound healing assays were further confirmed by performing a three-dimensional cell migration assay with the Transwell system (Fig. 2d). HeLa cells expressing full length gB did not migrate; instead, HeLa cells expressing gBΔR were found to migrate more efficiently (Fig. 2d). These data suggest the ability of gB lacking RGD to significantly accelerate motility of HeLa cells.

### A functional DLD within the gB is a necessity for the gBΔR to promote cell migration

**Fig. 3:**
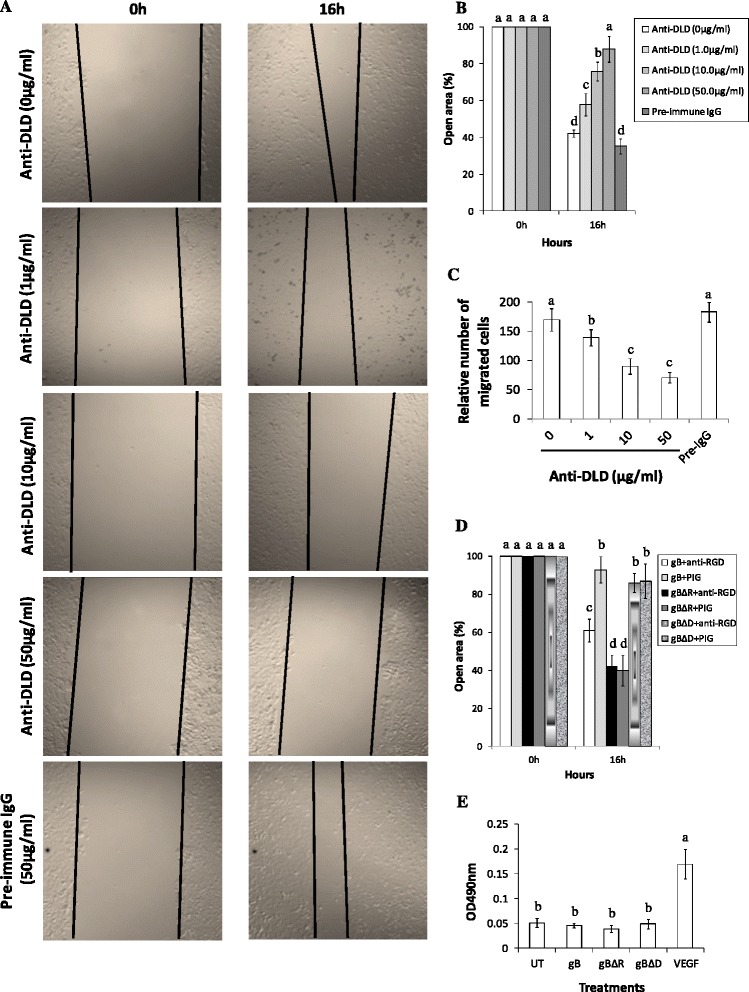
DLD of gB is critical to mediating cell migration. **a, b, d** HeLa cells stably expressing gB (**d**), gBΔR (**a, b, d**), or gBΔD (**d**) in 24 well plates when 80-90 % confluent were scratched with a 1000 μl pipette tip. Post scratch, the cells were incubated with medium supplemented with different concentrations of anti-DLD antibodies (**a, b**), anti-RGD antibodies (**d**) or pre-immune IgGs (PIG). Wound closure was monitored at 16 h post scratch and imaged with a laser-scanning LSM 510 Carl Zeiss confocal microscope (Magnification, x 20 objective). A representative image of cell migration is provided (**a**). **b, d** The open area (scratch) was quantified with TSratch software and the data represented as a histogram. **c** Data from Transwell migration assays showing alterations on the migration of HeLa cells expressing gBΔR through a permeable membrane in response to incubating them with anti-DLD antibodies is depicted. **e** MTT cell proliferation assay was performed to monitor the effect of gB on cells. Briefly, target cells were plated to a density of 3,000 cells/well in a 96-well plate in 150 μl volume of growth medium (DMEM supplemented with 10 % FBS). The following day, cells were incubated with DMEM containing 2 % FBS. As a positive control, we supplemented 10 ng/ml of VEGF to one of the wells seeded with untransfected cells. After 72 h, the MTT assay was performed. Each experiment was repeated three times. Columns with different alphabets (panels **b, c, d, and e**) indicate the values to be statistically significant (*p* < 0.05) by LSD

The gB lacking RGD but containing a fully functional DLD mediates cell migration. Hence, in this section we wanted to confirm the role for DLD in mediating migration of cells expressing gBΔR. This was done by using anti-DLD antibodies to test if it could block the migration of cells expressing gBΔR. Incubating cells with anti-DLD antibodies compared to pre-immune IgGs significantly lowered the ability of gBΔR to mediate migration of cells (Fig. 3a). We observed a dose dependent effect of anti-DLD antibodies on blocking migration of cells expressing gBΔR. Quantification of the wound closure is presented in the bar diagram in Fig. 3b. As expected, anti-DLD antibodies did not alter the migration of cells expressing gB (Additional file 1: Figure S2). Interestingly, 20 ng/ml of epidermal growth factor (EGF)-induced migration of HeLa cells could not be blocked by 50 μg/ml of anti-DLD antibodies suggesting the selectivity of the antibodies in blocking the cell migration (data not shown). The results from wound healing assays were further confirmed by performing a three-dimensional cell migration assay with the Transwell system (Fig. 3c). Incubating HeLa cells stably expressing gBΔR with anti-DLD antibodies significantly and specifically inhibited the ability of these cells to migrate. These data suggest a key role for DLD of gB to significantly accelerate motility of HeLa cells. To further authenticate the effect of DLD in assisting migration of cells, we tested the effect of incubating HeLa cells expressing gB, gBΔR, and gBΔD with 60 μg/ml of anti-RGD antibodies on migration. Anti-RGD antibodies significantly enhanced migration of cells expressing gB in contrast to cells expressing gBΔD (Fig. 3d). Anti-RGD antibodies compared to pre-immune IgGs did not amplify the cell migration pattern that was observed in cells expressing gBΔR (Fig. 3d). Taken together, a functional DLD in gB is critical to gBΔR-induced migration of cells. In order to confirm that the wound closure in cells expressing gBΔR is due to actual migration of cells and not cell proliferation, we monitored proliferation of cells in untransfected cells compared to cells expressing different forms of gB. We monitored cell proliferation by two different methods: (i) MTT cell proliferation assay and (ii) standard cell counting technique. Results from MTT assay (Fig. 3e) and the conventional cell counting technique (data not shown) indicated that the proliferation of cells was not significantly altered in cells expressing recombinant gB forms compared to treating cells with soluble recombinant vascular endothelial growth factor (VEGF). These results further confirm that the wound closure in cells expressing gBΔR on the surface of cell membrane is actually due to migration and not due to an increase in proliferation.

### KSHV gBΔR induces exaggerated changes on the cell surfaces

**Fig. 4:**
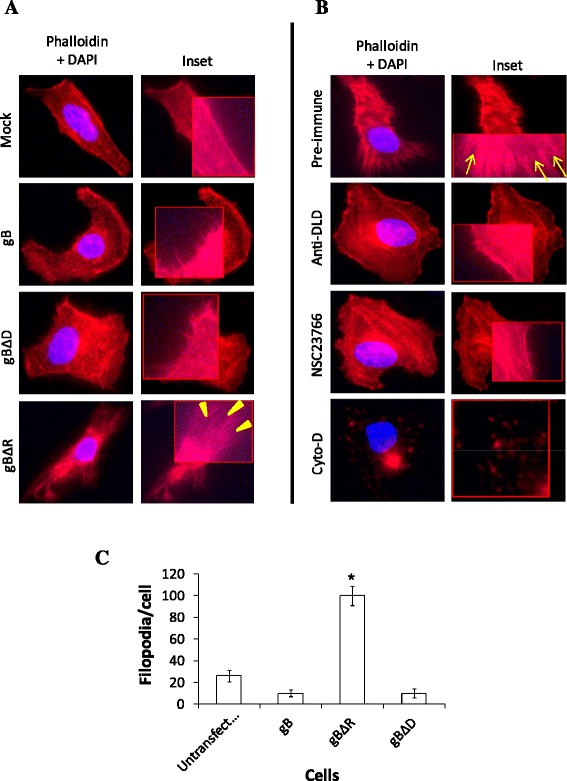
HeLa cells expressing gBΔR induce exaggerated changes at the edges of the cell membranes. **a** HeLa cells stably expressing gB, gBΔD, and gBΔR were fixed, permeabilized, and stained for polymerized actin by rhodamine-labeled phalloidin for 20 min at room temperature, washed, mounted using a mountant with DAPI prior to examining under a Nikon microscope using appropriate filters. **b** In a separate experiment, the cells expressing gBΔR were either treated with medium supplemented with 50 μg/ml of pre-immune IgGs or anti-DLD IgGs; or treated with 50 μM NSC23766 (Rac1 inhibitor) or 1 μM cyto-D for 2 h, prior to staining as above and viewing under the fluorescent microscope. A representative figure of the cells under different conditions is provided at a magnification of x 100. Closed yellow arrow heads, and arrows denote filopodial and lamelipodia, respectively. **c** Filopodial quantification: 300-500 μm long filopodial structures were counted on ten randomly selected cells for each treatment and the numbers were averaged. To avoid bias, the counting of filopodial projections for each treatment was performed by two different individuals and averaged. Each point on the plot represents the average from three different experiments. Asterisks on the data point denote the value to be statistically significant (*p*˂0.05) by LSD

Cell migration is a mechanically intensive cellular process that is mediated by the dynamic assembly and contractility of the actin cytoskeleton [35]. One of the hallmarks of cell migration is the reorganization and remodeling of actin cytoskeleton which are regulated by the Rho family of small GTPases, Rho, Rac, and cdc42 [36]. Rho, Rac, and cdc42 act in a well orchestrated manner to induce actin polymerization that leads to the formation of filopodia (protrusions), lamelipodia, and stress fibers that eventually result in the movement of cells [37]. Hence, we attempted to understand the activity in HeLa cells expressing gBΔR compared to cells expressing gBΔD and gB. Compared to mock transfected cells, cells expressing gB and gBΔD demonstrated filopodial extensions (Fig. 4a). However, such filopodial extensions were exaggerated multiple times along with the appearance of lamelipodia in cells expressing gBΔR on their cell surfaces (Fig. 4a, c). Interestingly, culturing these cells expressing gBΔR in medium supplemented with 50 μg/ml of anti-DLD IgGs significantly reduced the appearance of these membrane activities (Fig. 4b). A widely used Rac1 inhibitor, NSC23766 [38], significantly limited the appearances of filopodia and lamelipodia in HeLa cells expressing gBΔR (Fig. 4b). Treating HeLa cells expressing gBΔR with cytochalasin D (Cyto-D), a known inhibitor of actin dynamics, significantly lowered polymerization of actin and this was also used as a control. These results demonstrated a plausible cause (occurrence of dynamic events on the surface of cells) for the migration of cells expressing gBΔR compared to those expressing gB and gBΔD.

### Rac1 associated signaling is critical for gBΔR induced cell migration

**Fig. 5:**
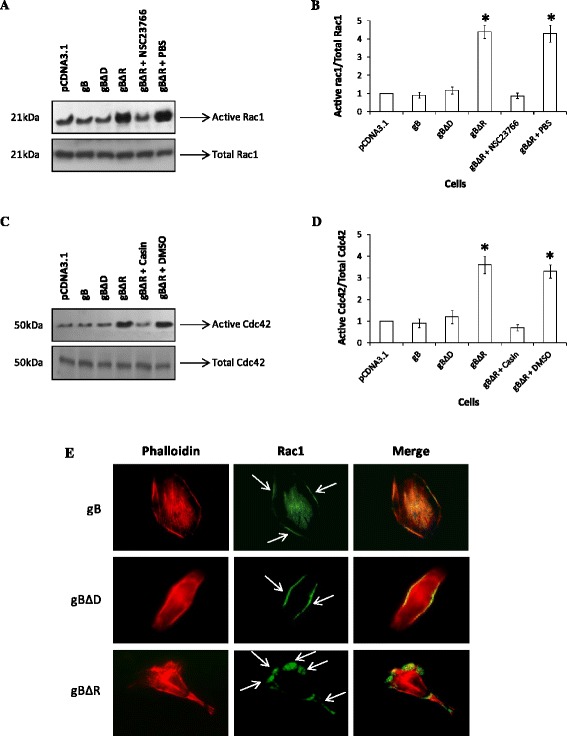
Rac1/Cdc42 signaling is induced in cells expressing gBR. Rac1 (**a, b**) and Cdc42 (**c, d**) activity was measured using GST-Pak1 pulldown assays in cells expressing gB, gBΔD, and gBΔR in the absence or in the presence of a NSC23766 (Rac1 inhibitor) and Casin (Cdc42 inhibitor). **b, d** The graph shows densitometric analysis of Rac/Cdc42-GTP levels with respect to input levels of total Rac1/Cdc42 for 5 independent experiments. **e** Cells expressing different forms of gB were permeabiized and double stained with anti-Rac1 (*Green*) and phalloidin (*Red*). The two images were merged to show the co-localization (denoted by arrows). Magnification: x100

**Fig. 6:**
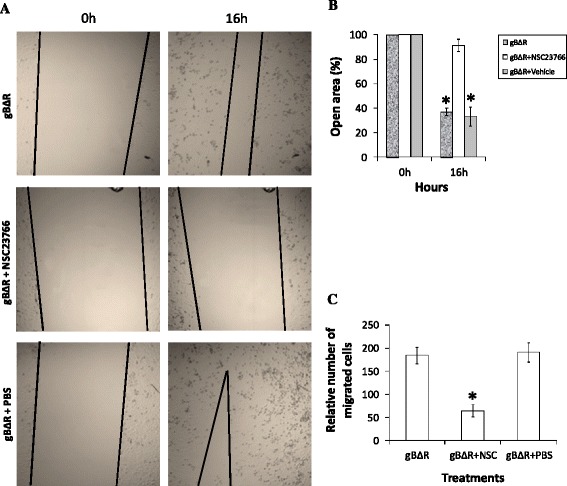
Rac1 signaling is critical for migration of cells expressing gBΔR. **a** HeLa cells stably expressing gBΔR in 24 well plates when 80-90 % confluent were scratched with a 1000 μl pipette tip. After the scratch was performed, the cells were incubated with medium or medium supplemented with 50 μM of NSC23766 or vehicle (PBS). Wound closure was monitored at 16 h post scratch and imaged using a confocal microscope (Magnification, x 20 objective). A representative image of cell migration is provided. **b** The open area (scratch) was quantified with TSratch software and the data represented as a histogram. **c** Data from Transwell migration assays showing alterations on the migration of HeLa cells expressing gBΔR through a permeable membrane in response to incubating them with Rac1 inhibitor and the vehicle for the inhibitor (PBS) is depicted. Columns with asterisk mark (panels **b** and **c**) denotes the value to be statistically significant (*p* < 0.05) by LSD

Rac1 and Cdc42 are among the main driving forces in the formation of lamelipodial and filopodial extensions [39–41]. These findings prompted us to investigate whether Rac1 and Cdc42 do play a critical role in gBΔR mediated cell migration. We first analyzed the manner in which Rac1 and Cdc42 expression varied in HeLa cells expressing gB, gBΔD, and gBΔR using a Rac1 and Cdc42 pull down assay. The pull down assays demonstrated significantly elevated levels of Rac1 and Cdc42 activity in cells expressing gBΔR compared to cells expressing gB, or gBΔD (Fig. 5a, c). The total Rac1 and Cdc42 levels in all of the treatments were comparable (Fig. 5a, c). The elevated levels of Rac1 and Cdc42 activity in cells expressing gBΔR was significantly lowered in cells treated with Rac1 inhibitor (NSC23766) and Cdc42 inhibitor (Casin) when compared to cells that were treated with PBS or DMSO, the respective vehicles for the inhibitors (Fig. 5a-d). Between Rac1 and Cdc42, the former has a more crucial role in mediating migration of cells [42] and hence this study focused more on Rac1 associated signaling than Cdc42. Distribution of active Rac1 was observed at the leading edges of the cells to regulate localized actin polymerization and membrane protrusions irrespective of the cells (expressing gB, gBΔD, or gBΔR). However, the expression levels of Rac1 were pronounced in cells expressing gBΔR compared to gB, gBΔD (Fig. 5e), and empty vector (data not shown). We then compared the ability of the Rac1 inhibitor to block the migration of cells induced by surface expression of gBΔR by employing the wound healing assays. Our results demonstrated Rac1 inhibitor to significantly block the ability of gBΔR to mediate cell migration (Fig. 6a). The Rac1 inhibitor did not alter the ability of gB to mediate cell migration (Additional file 1: Figure S3). Quantification of the wound closure is represented by the bar diagram in Fig. 6b. The results from wound healing assays were further confirmed by performing a three-dimensional cell migration assay with the Transwell system (Fig. 6c). Incubating HeLa cells expressing gBΔR with Rac1 inhibitor significantly inhibited the ability of these cells to migrate compared to incubating cells with the vehicle for the Rac1 inhibitor, PBS. We observed identical results when Cdc42 inhibitor was tested (data not shown). These data suggest a key role for Rac1 and Cdc42 associated signaling in the ability of gBΔR to accelerate motility of HeLa cells.

### Silencing the upstream effector of Rac1 signaling significantly blocks migration of cells expressing gBΔR

**Fig. 7:**
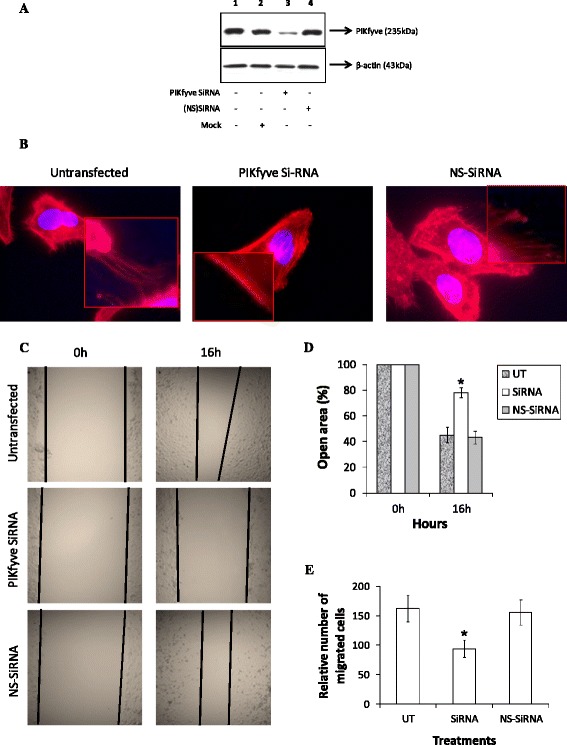
PIKfyve is involved in migration of cells expressing gBΔR. **a** Western blotting analysis demonstrating the efficient decrease in PIKfyve protein levels after SiRNA transfection. **b** Fluorescent imaging demonstrating the dynamic changes on the surfaces of stably transfected HeLa cells expressing gBΔR but transfected with PIKfyve SiRNA or NS-SiRNA compared to mock transfected cells. A representative figure of the cells under different conditions is provided at a magnification of x100. Yellow lines denote the length and the area covered by filopodial extensions. **c** The migration assay was performed in five independent experiments using cells that were either mock transfected, PIKfyve SiRNA, or NS-SiRNA transfected. **d** The open area (scratch) was quantified with TSratch software and the data represented as a histogram. **e** Data from Transwell migration assays showing alterations on the migration of HeLa cells expressing gBΔR that were transfected with PIKfyve SiRNA or NS-SiRNA is depicted. Columns with asterisk mark (panels **d** and **e**) denotes the value to be statistically significant (*p* < 0.05) by LSD

Recent studies demonstrated phosphoinositide 5-kinase, FYVE finger containing enzyme, PIKfyve, as one of the upstream molecules known to regulate migration of cells by activating Rac1 [43]. In order to further confirm a role for Rac1 associated signaling, we attempted to analyze the ability of cells expressing gBΔR to migrate under conditions where the expression of PIKfyve was silenced by using specific SiRNA. For this purpose, we used HeLa cells stably expressing gBΔR (Fig. 2a). These cells expressing gBΔR were untransfected, mock transfected, transfected with PIKfyve SiRNA, or NS-SiRNA. At 48 h post transfection of SiRNA, cells were analyzed for the expression of PIKfyve by Western blotting using specific antibodies. Transfection of HeLa cells expressing gBΔR with PIKfyve SiRNA significantly reduced the expression of PIKfyve (Fig. 7a). Mock transfection or transfection of HeLa cells expressing gBΔR with NS-SiRNA did not alter the expression of PIKfyve (Fig. 7a). The effect of NS-SiRNA on PIKfyve expression was comparable to what was observed in untransfected cells (Fig. 7a). These cells were analyzed by staining for F-actin using rhodamine-labeled phalloidin. HeLa cells expressing gBΔR that were either untransfected or transfected with NS-SiRNA demonstrated significant amounts of dynamic changes on the cell membranes including long filopodial extensions while, PIKfyve SiRNA transfected cells expressing gBΔR only had limited filopodial extensions (Fig. 7b). We then compared the ability of silencing PIKfyve to block the migration of cells induced by surface expression of gBΔR. Our results demonstrated knocking down PIKfyve significantly blocked the ability of gBΔR to mediate cell migration (Fig. 7c). Silencing PIKfyve did not alter the ability of gB to mediate cell migration (Additional file 1: Figure S4). Mock transfection or transfection of cells with NS-SiRNA did not alter the ability of cells expressing gBΔR to migrate. Quantification of the wound closure is represented by the bar diagram in Fig. 7d. The results from wound healing assays were further confirmed by performing a three-dimensional cell migration assay with the Transwell system (Fig. 7e). HeLa cells expressing gBΔR and those transfected with NS-SiRNA were found to migrate more efficiently compared to cells transfected with PIKfyve SiRNA (Fig. 7e). These data suggest a key role for PIKfyve > Rac1 associated signaling in the ability of gBΔR to accelerate motility of HeLa cells.

### RGD is required for cell attachment

**Fig. 8:**
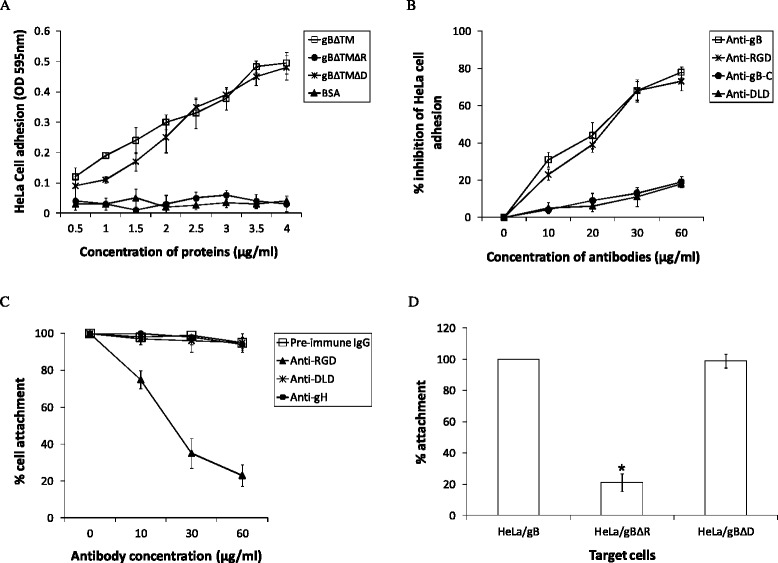
RGD of gB is required for attachment of cells. **a** KSHV gBΔTM protein-induced cell adhesion. Maxisorp plates were coated with different concentrations of purified gBΔTM, gBΔTMΔR, gBΔTMΔD, or BSA proteins overnight at 4 °C, washed, and cell adhesion assays were carried out. Each point on the plot represents the average from five experiments. **b** Antibodies to RGD inhibit the HeLa cell adhesion to gBΔTM protein. Maxisorp plates were coated with 4 μg of purified gBΔTM protein/ml overnight at 4 °C, washed, and incubated for 1 h at 4 °C with different antibodies prior to seeding the plate with HeLa cells and performing the cell adhesion assay. Data are presented as percentage of inhibition of cell adhesion obtained when gBΔTM was preincubated with DMEM only. Each point on the plot represents the average from five experiments. **c** Anti-RGD antibodies block the ability of gB expressed on the surfaces of HeLa cells to promote cell adhesion. HeLa cells expressing full length gB were incubated with various antibodies for 1 h at +4 °C prior to performing cell adhesion assay in a 24-well plate. Data are presented as percentage cell adhesion observed when cells expressing gB were preincubated with DMEM only. **d** gBΔR does not promote cell adhesion. HeLa cells expressing gB, gBΔR, and gBΔD were tested in adhesion assay. Data are presented as percentage cell attachment observed when cells expressing gB were tested. Each point on the plot represents the average from five experiments. Column with asterisk mark denotes the value to be statistically significant (*p* < 0.05) by LSD

Our earlier studies utilized cell adhesion assays effectively by employing soluble gB (gBΔTM) and gBΔTM lacking a functional RGD (gBΔTMΔR) to decipher a critical role for the RGD domain in promoting cell adhesion [21]. In the present study, we analyzed the role of DLD contained within the gB to promote adhesion of cells by employing and comparing the effect of gBΔTMΔD [10] with gBΔTM [10] and gBΔTMΔR [21]. Our studies demonstrated gBΔTM and gBΔTMΔD to significantly mediate adhesion of HeLa cells when compared to gBΔTMΔR and BSA (Fig. 8a).

To ascertain the specificity of HeLa cell adhesion, gBΔTM-coated plates were preincubated with different concentrations of IgG antibodies for 1 h at 4 °C before seeding of HeLa cells for adhesion assays. Rabbit anti-gB antibodies and anti-RGD antibodies significantly inhibited the gBΔTM mediated adhesion of HeLa cells (Fig. 8b). In contrast, rabbit antibodies against the gB peptide lacking the RGD motif but with RGY amino acids (anti-gB-C antibodies) and anti-DLD antibodies did not show any significant inhibition of cell adhesion (Fig. 8b). These results suggested a critical role for the RGD and not the DLD domain of gB to mediate adhesion of HeLa cells.

To authenticate these results in cells expressing gB on their surfaces, we used HeLa cells stably expressing different forms of gB on the cell surfaces (Fig. 2a). In the first experiment, we employed HeLa cells expressing gB in adhesion assay. Our results demonstrated anti-RGD antibodies to specifically block the adhesion of HeLa cells expressing gB compared to anti-DLD antibodies, anti-gH antibodies, or pre-immune IgGs (Fig. 8c). In the next experiment, we employed HeLa cells expressing gB, gBΔR, and gBΔD on the surface of cells. These cells were tested for their ability to attach. There was a significant drop in the ability of HeLa cells expressing gBΔR to adhere compared to cells expressing gB and gBΔD (Fig. 8d). Taken together, we conclude that the RGD domain (and not the DLD) in gB is a necessity for mediating adhesion of cells.

## Discussion

Cell migration requires a coordinated series of events including cell adhesion, detachment of adhesions, reorganization of the actin cytoskeleton, and membrane protrusion and retraction [44]. The balance between cell adhesion and detachment of adhesions is very important for cell migration [45]. Our earlier studies identified a role for RGD domain in gB interactions with αV integrins to enhance attachment of cells [12]. Taken together, we predict gB blocks cell migration in an RGD-dependent manner by actually increasing the cell adhesion; thus preventing cells from migrating. These functional findings are identical to what has been reported in a disintegrin and metalloprotease protein family-15 (ADAM15) which, though possessing both RGD and DLD, inhibits integrin-dependent cell migration in an RGD-dependent manner [46, 47].

KSHV gB may well be a functional homologue of the cellular ADAM15. ADAM15 is the only ADAM member that contains both RGD and DLD [46]. The one minor difference between ADAM15 and gB is that ADAM15 contains RGD within its DLD while in the case of gB, both RGD and DLD are distinctly separate domains. Accordingly, in this study we used gB that contains two distinct integrin binding domains (RGD and DLD) to determine how these interactions alter the ability of a protein to influence attachment and migration of cells.

When the first study described KSHV gB to possess a RGD domain [19], we hypothesized gB to be a multi-functional protein. It was based on the following facts: (i) gB is expressed on the virus envelope and on the surface of infected cell membranes; (ii) gB interacted with integrins via its RGD domain; and (iii) integrins regulate a variety of cellular functions including cell adhesion, migration, angiogenesis, and cancer progression [48, 49]. Our studies determined gB to not only aid KSHV binding [11] and entry [19] into cells but also mediate attachment of cells [12]. We were aware of the fact that RGD and DLD domains have opposing effects [7, 50, 51]. Hence, we tested different recombinant gB proteins on their ability to support migration of cells. In the process, we tested full length gB instead of using soluble form of the proteins.

Our results demonstrated gB lacking a functional RGD domain (gBΔR) compared to the wild-type gB or gBΔD to promote migration of cells (Fig. 2). Interestingly, cell migration induced by gB lacking a functional RGD required a functional DLD domain (Fig. 3). Cell migration is dependent on dynamic events like formation of filopodia and lamellipodia that occur on the edges of the cell membrane [52]. In this study, we determined cells expressing gBΔR to demonstrate exaggerated filopodial extensions and formation of lamellipodia that could be specifically inhibited by Rac1 inhibitor or by blocking the DLD functions using anti-DLD IgGs (Fig. 4). The results from the present study differed from an earlier study which described gB to induce filopodia and lamellipodia [53]. This discrepancy may be due to the fact that in the earlier study, soluble form of gB was used compared to the physiologically relevant full length gB tested in this study. However, the results from this study do not in any way refute the conclusions drawn using the soluble form of gB. Overall, the dynamic events in terms of actin polymerization occurring on the surfaces of the membrane are critical to supporting migration induced by gBΔR.

One of the major changes that were observed in cells expressing gBΔR was the presence of long filopodial extensions and lamellipodia. Filopodial extensions and lamellipodia are induced by Rac1 and cdc42-associated signaling [35]. Active forms of Rac1 and Cdc42 were elevated in cells expressing gBΔR compared to those expressing gB, and gBΔD (Fig. 5). It is generally understood that Rac1 would activate PAK, a kinase known to phosphorylate the specific guanine nucleotide exchange factor of Cdc42. Cdc42, in turn, will activate effectors such as WAS, WASP or ERK, involved in the organization of filopodia and lamellipodia [54]. Hence, we analyzed the effect of Rac1 inhibitor on the ensuing migration of cells. Rac1 inhibitor lowered Rac1 activity in cells expressing gBΔR and thus the migration of those cells (Fig. 6). Recent studies also identified a critical role for PIKfyve in inducing migration of cells [43]. Silencing PIKfyve expression in cells specifically expressing gBΔR using SiRNA significantly inhibited expression of PIKfyve, filopodia and lamellipodia, and migration of cells (Fig. 7). Transfection of cells with NS-SiRNA did not significantly alter the expression of PIKfyve, filopodia and lamellipodia, and migration of cells. Based on these results, we concluded the following about the RGD and DLD domains: (i) DLD domain in gB (in the absence of RGD) induces migration of cells via Rac1 and PIKfyve associated cell signaling; (ii) RGD domain in gB is the switch that turns off the effect of DLD in inducing migration of cells. In this study, we have closely monitored the effect of cell surface expressed different forms of gB in regulating migration versus attachment of cells. Our future studies will focus on delineating the paracrine effects of gB-induced growth factor(s) in preferentially mediating cell attachment over migration.

The final question asked was, does DLD domain in gB have any role to play in promoting adhesion of cells? Our results clearly implicate a null role for DLD in mediating cell attachment (Fig. 8). Attachment of cells is strictly mediated by the RGD domain as described in earlier studies [21].

The study also provides a glimpse into how the RGD and DLD domains function as a unit to affect function of gB. Future studies are underway to understand the manner by which DLD and RGD-based interactions alter RhoA, Rac1, and cdc42 in virtually promoting attachment of cells over migration. This study puts forth critical questions to better understand the manner by which RGD domain regulates the function of DLD within a protein in regulating migration of cells: (i) How does DLD alter cellular signaling to promote migration of cells; and (ii) How does RGD-induced cellular signaling promote attachment of cells over DLD-induced migration of cells?

## Conclusion

The significance of the present study is that it demarcates the roles of two distinct integrin binding domains contained within KSHV gB in modulating attachment and migration of cells. Based on our earlier and present studies, we propose a model where we believe RGD-based integrin interactions that stimulate focal adhesion kinase (FAK) activity are critical to promoting adhesion of cells [21] while DLD-based integrin interactions that stimulate Rac1 are crucial in promoting migration of cells (Table 1). Increase in FAK activity has an inverse correlation with migration of cells [55]. In terms of KSHV pathogenesis, gB, that possesses both the RGD and DLD-integrin binding domains actually promote attachment of cells compared to migration. In a way, late structural protein gB mediates attachment of cells thereby supporting a lytic phase of viral replication as suggested in an earlier study [12] with a limited role in directly promoting cancer development.

**Table 1:** Correlation between attachment and migration of cells to FAK and Rac1 activity

Protein	gB	gBΔD	gBΔR	Function
FAK	+++	+++	+	Attachment
Rac1	+	+	+++	Migration

## Additional file

Additional file 1:
**Supplementary section.** (PDF 574 kb)

## Abbreviations

RGD: Arg-Gly-Asp
DLD: disintegrin-like domain
gBΔR: gB lacking a functional RGD
gBΔD: gB lacking a functionally intact DLD
SiRNA: small interfering RNA
KSHV: Kaposi’s sarcoma-associated herpesvirus
