# Supplementary material for: KSHV gB associated RGD interactions promote attachment of cells by inhibiting the potential migratory signals induced by the disintegrin-like domain
Source: BMC Cancer. 2016 Feb 24;16:148. doi: 10.1186/s12885-016-2173-9 (PMC4766674; doi:10.1186/s12885-016-2173-9)
Supplement: Additional file 1: — Supplementary section. (PDF 574 kb) [file 12885_2016_2173_MOESM1_ESM.pdf]

SUPPLEMENTARY SECTION:

FIGURES:

SFigure 1a:

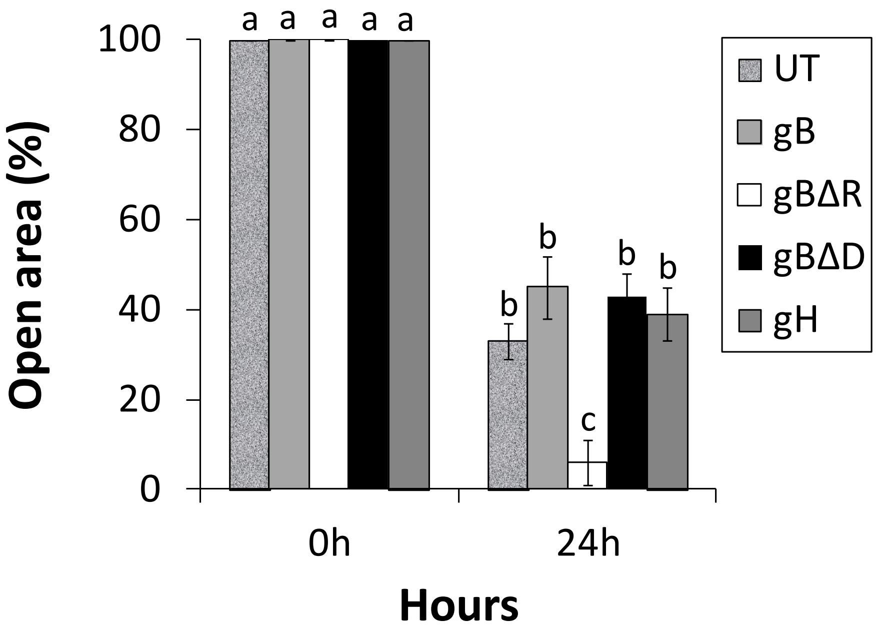

SFig. 1a: Expression of gBΔR accelerates cell migration in wound healing assays. Untransfected HeLa cells or cells expressing gB, gBΔD, gBΔR, and gH in 24 well plates when 80-90% confluent were scratched with a 1000µl pipette tip. Wound closure was monitored at 24h post scratch. The open area (scratch) was quantified with TSratch software and the data represented as a histogram. The experiment was repeated three times. Columns with different alphabets indicate the values to be statistically significant ($p<0.05$) by LSD.

SFigure 1b:

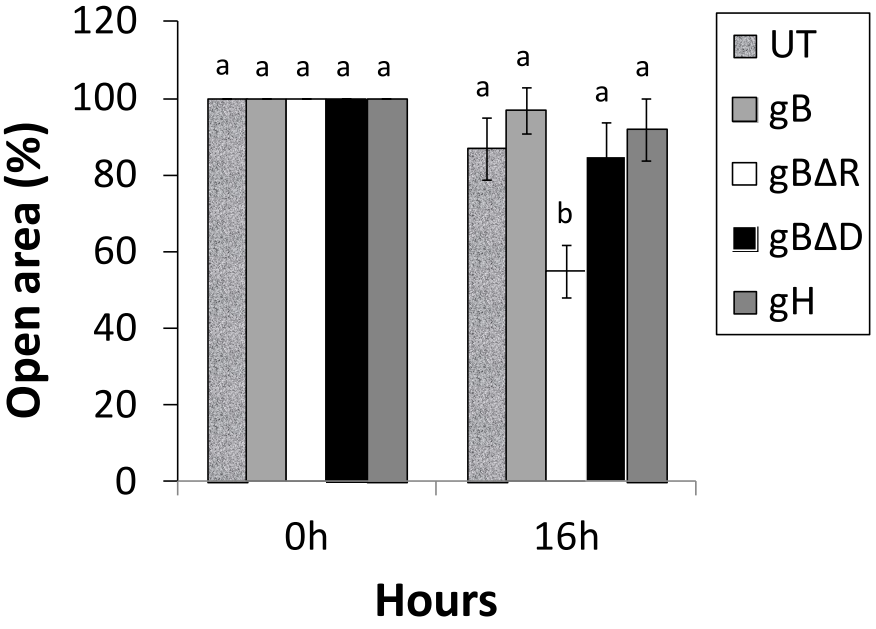

SFig. 1b: Expression of gBΔR accelerates HUVEC cell migration in wound healing assays. Untransfected HUVEC cells or cells transiently expressing gB, gBΔD, gBΔR, and gH in 24 well plates when 80-90% confluent were scratched with a 1000μl pipette tip. Wound closure was monitored at 16h post scratch. The open area (scratch) was quantified with TSratch software and the data represented as a histogram. The experiment was repeated three times. Columns with different alphabets indicate the values to be statistically significant ($p<0.05$) by LSD.

SFigure 2:

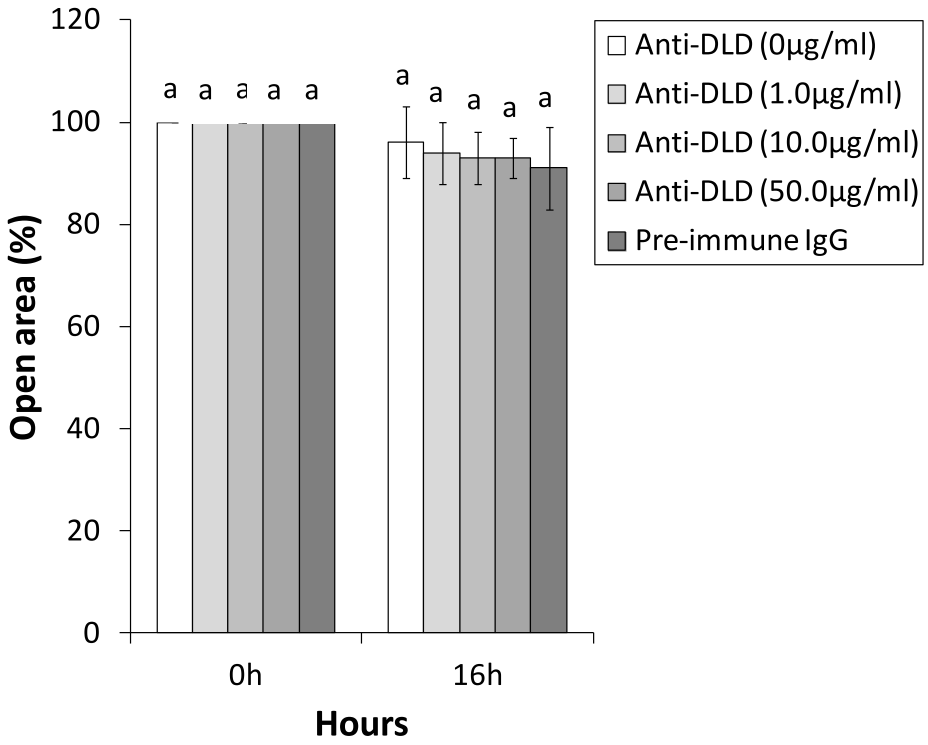

SFig. 2: Anti-DLD antibodies do not alter migration of cells expressing gB. HeLa cells stably expressing gB in 24 well plates when 80-90% confluent were scratched with a 1000μl pipette tip. Post scratch, the cells were incubated with medium supplemented with different concentrations of anti-DLD antibodies or pre-immune IgGs. Wound closure was monitored at 16h post scratch and imaged with a laser-scanning LSM 510 Carl Zeiss confocal microscope (Magnification, x 20 objective). The open area (scratch) was quantified with TSratch software and the data represented as a histogram. Each experiment was repeated three times. Columns with different alphabets indicate the values to be statistically significant ($p<0.05$) by LSD.

SFigure 3:

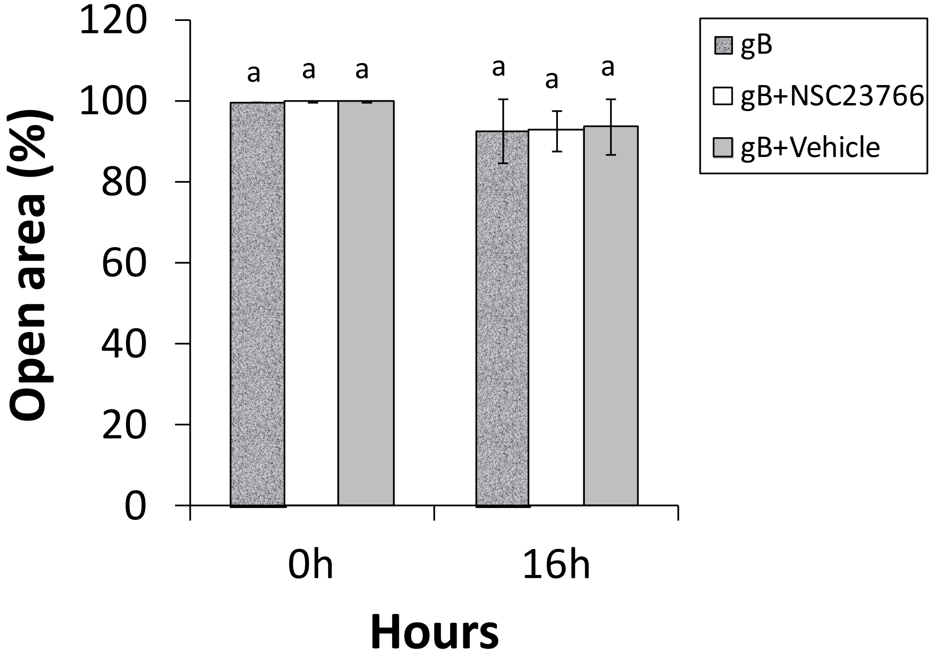

SFig. 3: Rac1 inhibitor does not alter the migration pattern of cells expressing gB. HeLa cells stably expressing gB in 24 well plates when 80-90% confluent were scratched with a 1000µl pipette tip. After the scratch was performed, the cells were incubated with medium or medium supplemented with 50µM of NSC23766 or vehicle (PBS). Wound closure was monitored at 16h post scratch and imaged using a confocal microscope (Magnification, x 20 objective). The open area (scratch) was quantified with TSratch software and the data represented as a histogram. Each experiment was repeated three times. Columns with different alphabets indicate the values to be statistically significant ($p<0.05$) by LSD.

SFigure 4:

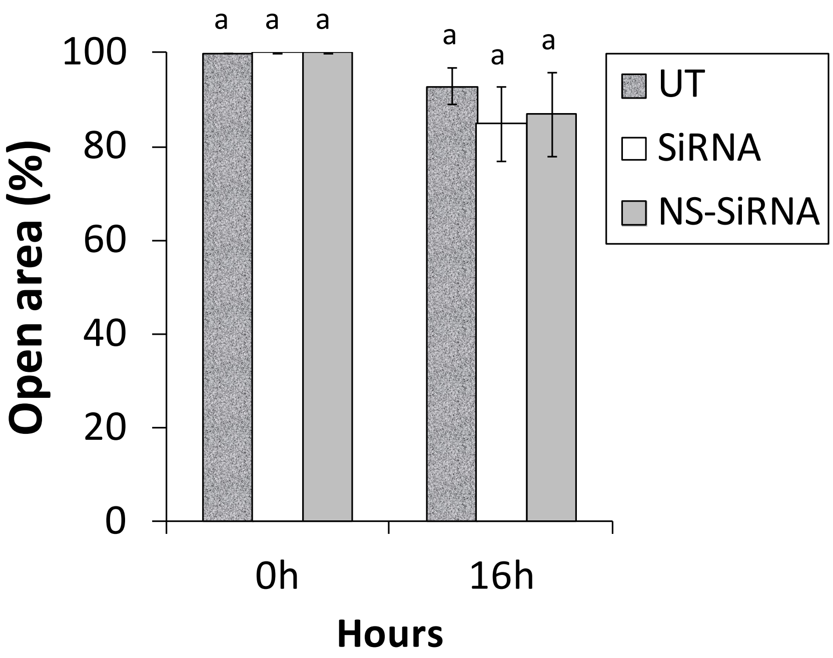

SFig. 4: PIKfyve SiRNA failed to alter the migration pattern of cells expressing gB. The migration assay was performed in three independent experiments using cells expressing gB that were either mock transfected, PIKfyve SiRNA, or NS-SiRNA transfected. Columns with different alphabets indicate the values to be statistically significant ($p<0.05$) by LSD.
